# Phytochemical screening and biological evaluation of Greek sage (*Salvia fruticosa* Mill.) extracts

**DOI:** 10.1038/s41598-023-49695-w

**Published:** 2023-12-15

**Authors:** Marika Mróz, Barbara Kusznierewicz

**Affiliations:** Department of Chemistry, Technology and Biotechnology of Food, Faculty of Chemistry, Gdańsk University of Technology, Narutowicza 11/12 St., 80-233 Gdańsk, Poland

**Keywords:** Mass spectrometry, Nutritional supplements

## Abstract

This study explores the influence of extraction solvents on the composition and bioactivity of *Salvia fruticosa* extracts. Ultrasound-assisted extraction with water, ethanol and their mixtures in variable proportions was used to produce four different extracts. An untargeted UPLC/MS‑based metabolomics was performed to discover metabolites profile variation between the extracts. In the analyzed samples, 2704 features had been detected, of which 95 were tentatively identified. The concentrations of the important metabolites, namely, caffeic acid, carnosic acid, carnosol, rosmarinic acid, salvianolic acid B and scutellarin, were determined, using UPLC-PDA methods. Rosmarinic acid was the dominant metabolite and antioxidant in all tested extracts, except the aqueous extract, in which scutellarin was the most abundant compound. The extracts and standards were examined for antioxidant activity and xanthine oxidase (XO) inhibitory activity. The most diverse in terms of chemical composition and rich in antioxidant compounds was 70% ethanolic extract and the strongest antioxidant was caffeic acid. All analyzed extracts showed the ability to inhibit XO activity, but the highest value was recorded for 30% ethanolic extract. Among tested standards, the most potent XO inhibitor was caffeic acid. The results suggest that the leaves of Greek sage are a source of natural XO inhibitors and may be an alternative to drugs produced by chemical synthesis.

## Introduction

Nowadays, the understanding of food extends far beyond its nutritional value. Apart from the taste sensations, the use of herbs is heading towards enrichment of the diet with bioactive compounds. In the field of functional food additives or dietary supplements the chemical composition is of great importance. In addition to the characteristics of the plant material, the key factor in obtaining extracts with specific desired properties is the processing conditions. The application of novel analytical approaches such as metabolomics makes it possible to compare extraction efficiency by tracking multiple phytochemicals related to the quality and health-promoting properties of extracts simultaneously^1^.

*Salvia fruticosa* Mill. (syn. *S. triloba* L.f.) is a species of Mediterranean herb in *Lamiaceae* family. *S. fruticosa* has been used in many forms as a traditional medicine for different purposes depending on the region. In Lebanon, it was typically used as a remedy for ulcer pain and in Turkey, for urinary system ailments^2^. In the Mediterranean region *S. fruticosa* is considered even more valuable than *S. officinalis* in medicinal applications, owing to the richness of its essential oils^3^. The essential oils derived from Greek sage were proven to have antioxidant, antibacterial and anti-inflammatory activities and were used traditionally for treating skin infections^4,5^. The anti-inflammatory activity of *Salvia* species is also attributed to the presence of tanshinones, phenolics and flavonoids. Studies on *S. miltiorrhiza* indicated the anti-inflammatory activity of salvianolic acid B, tanshinone IIA and protocatechuic acid. In the case of *S. lavandulifolia* this activity was attributed to rosmarinic acid, genkwanin, luteolin, cirsimaritin, salvigenin, and also monoterpenes such as carvacrol and α-pinene^6^. In vivo studies on rats proved an antiobesity effect of *S. triloba* methanolic extracts through the inhibition of pancreatic triacylglycerol lipase and antineurodegenerative effect by improving biochemical and histopathological characteristics in Alzheimer's disease-induced rats^7,8^. Biological activities of herbal preparations depend greatly on their chemical composition. *S. triloba* essential oils are composed mainly of bicyclic oxygenated monoterpenes, e.g. 1,8-cineol, camphor and bicyclic hydrocarbon monoterpenes such as α- and β-pinene^9^, the infusions are abundant in polyphenols and flavonoids while alcoholic extracts are rich in di- and triterpenoids. Torun et al.^10^ studied the impact of water extraction at 60–80 °C on soluble solids, total phenolics and total flavonoids from *S. fruticosa* leaves harvested in the West Mediterranean Region of Turkey. In the available English-language literature, no detailed chemical composition of *S. triloba* infusion has been reported. According to studies on other *Salvia* species infusions, flavonoids content often exceed that of phenolic acids. The major constituents of the most popular sage species – *S. officinalis* infusions are luteolin 7-O-glucuronide and rosmarinic acid^11^. Methanolic sage extracts are rich in terpenoids, especially abietane diterpenes and triterpenes such as: carnosol, carnosic acid and ursolic acid. These compounds show strong antioxidant and anti-diabetic properties^12–14^.

The aim of the study was to examine the effects of using four different non-toxic solvents for ultrasound assisted extraction (UAE) of *S. fruticosa* to obtain extracts with certain biological activities. Therefore, the extracts were studied by means of chemical composition to find the most plentiful with known bioactives and additionally six compounds were selected representing the major constituents of studied *S. fruticosa* extracts to elucidate their correlation with antioxidant and xanthine oxidase inhibitory activity. Both biological activities were tested in vitro. Basic spectrophotometric methods of determining the antioxidant activity were supported by HPLC post-column derivatization with ABTS to provide a detailed analysis of the antioxidants present in studied *S. fruticosa* extracts. That allowed for indicating individual compounds with antioxidant properties present in the extracts. Owing to the complexity of herbal extracts, some components may interfere with typical spectrophotometric measurements of biological activity. Hence, to avoid such distortions, a new method of determining the xanthine oxidase (XO) inhibitory activity involving HPLC analysis is proposed in this study. Such detailed metabolomic studies play an important role in designing novel functional food or dietary supplements based on plant extracts, especially when targeting specific compounds. Although several review articles and reports on the chemical composition of *S. fruticosa* extracts are available, they usually concern the content and composition of phenolics. This study extends the current state of knowledge about the chemical composition of Greek sage, while providing important information on the bioactivity of selected compounds commonly found in the *Lamiaceae* family.

## Results and discussion

### Metabolomic profiling using LC-Q-Orbitrap HRMS

In general, metabolomic studies on *Salvia* species in negative ionization mode tend to be more efficient than these in positive mode^15^. In this study, MS data analysis included the use of online and local databases provided by Compound Discoverer 2.1 software. Additionally, data collected from previous metabolomic studies on *Salvia* species were collected and merged into a mass list to be implemented as a local database. A total of 2704 substance peaks had been detected in *S. fruticosa* extracts in negative ion mode analysis. After filtering out the minor signals (Area < 10^4^), there were 98 metabolites of which 95 were tentatively identified as shown in Supplementary Table S1.

**Figure 1:**
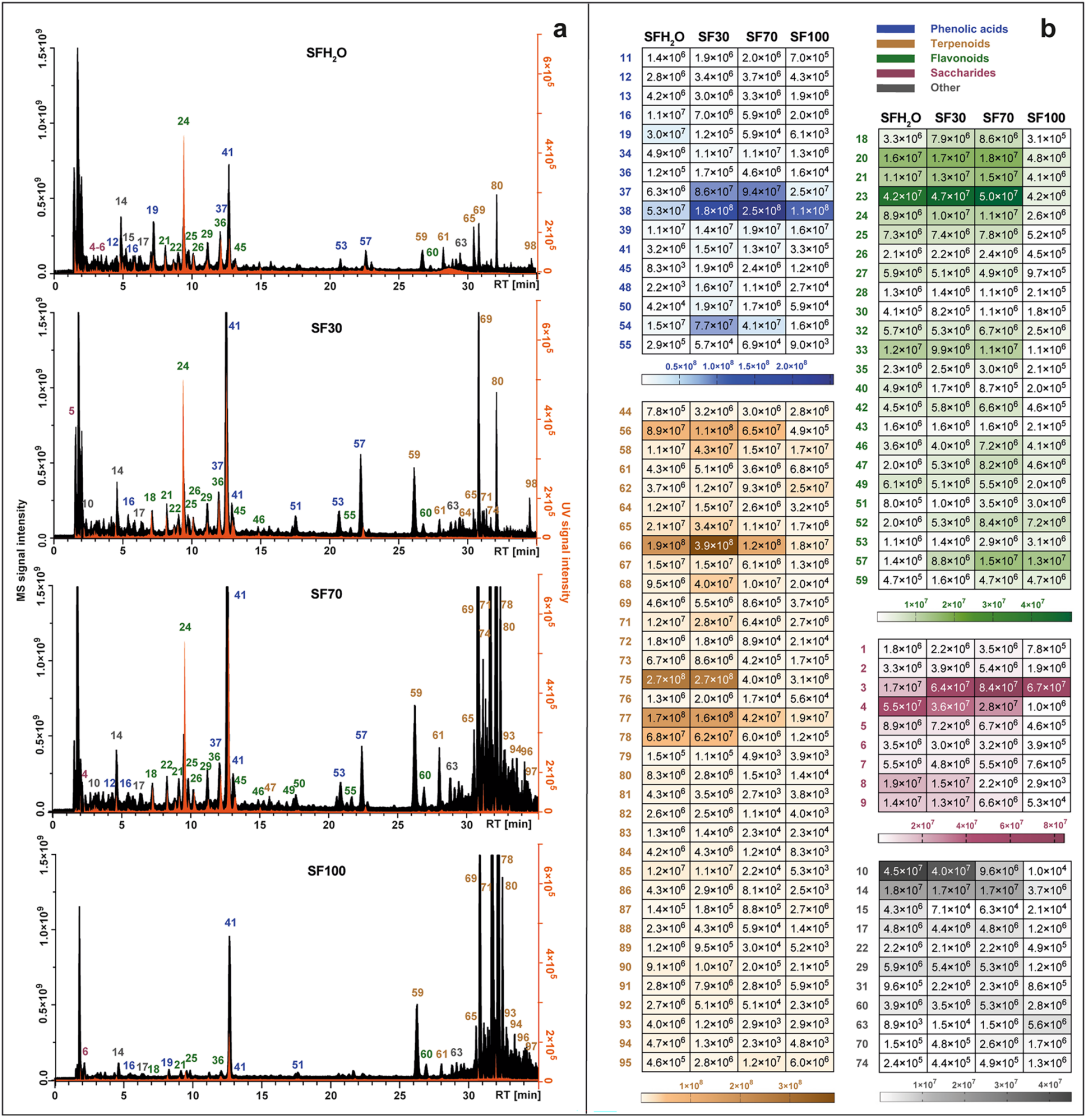
Total ion chromatograms obtained by LC-Q-Orbitrap in negative mode (black) combined with chromatograms registered by UV–Vis detector at 270 nm (orange) (**a**), set with heat map representing the mean MS peak area value of the identified compounds in four different *S. fruticosa* extracts: SFH_2_O–water extract; SF30–30% ethanol extract; SF70–70% ethanol extract; SF100–ethanol extract (**b**). For the identity of peaks, see Supplementary Table S1.

Metabolite profiles of each extract were juxtaposed and presented in Fig. 1a. The dominant groups changed among different extracts. In those obtained with solvents containing mostly water (SFH_2_O and SF30) the most abundant compounds were phenolic acids. The extraction efficiency of terpenoid compounds was consistent with increase of ethanol in used solvent owing to the non-polar nature of these compounds. Additionally, a heat map with the signal intensity of individual phytochemicals detected in four different *S. fruticosa* extracts is presented in Fig. 1b. The most numerous class of compounds detected in studied extracts was terpenoids with 35 compounds, followed by flavonoids (24 compounds), phenolic acids and derivatives (19 compounds), saccharides (9 compounds) and other such as fatty acids, carboxylic acids and unidentified compounds.

In the case of two most polar extracts (SFH_2_O and SF30) the phenolic acids were the most abundant classes in total peak area. This class was represented mainly by caffeic acid derivatives. The retention time (RT) of compound **16** with precursor ion [M-H]¯ at *m/z* 179.03419 was in line with the RT of the caffeic acid standard. It also generated characteristic major fragment at *m/z* 135.04414, due to loss of carbon dioxide. The deprotonated form of caffeic acid was detected in compounds **40** and **41**, which were identified as sagerinic acid ([M-H]¯ at *m/z* 719.16210) and rosmarinic acid ([M-H]¯ at *m/z* 359.0773). Rosmarinic acid identification was additionally confirmed by comparison with the standard. The same ion or its loss had been observed for compounds **20**, **37**, **39**, **44**, **53**, **57** and **58**, which supported by comparison with the literature and MS^2^ fragmentation, were identified as salviaflaside ([M-H]¯ *m/z* 521.13012), salvianolic acid B ([M-H]¯ at *m/z* 717.14661), isosalvianolic acid B ([M-H]¯ at *m/z* 717.14667), salvianolic acid K ([M-H]¯ at *m/z* 555.11469), two salvianolic acid F isomers ([M-H]¯ at *m/z* 313.07205) and salvianolic acid C ([M-H]¯ at *m/z* 491.09863).

In the case of the two most non-polar extracts (SF70, SF100) the contribution of terpenoids was the highest, while in the remaining extracts this class accounted for about a quarter of the sum of the peak area of the identified compounds. This class was represented mainly by diterpenoids, which were the most varied non-polar class of compounds identified in studied extracts. They were mostly abietane-type diterpenoids, for which fragmentation through negative ionization oftentimes included the removal of CO_2_ (-44 Da), CO (-28 Da), H_2_O (-18 Da), ·CH_3_ (15 Da). Compounds **59** ([M-H]¯ at *m/z* 345.17075) and **64** ([M-H]¯ at *m/z* 345.17100) both displayed ions attributable to the loss of carbon dioxide molecule (*m/z* 301.18097) and water molecule (*m/z* 283.17038 and *m/z* 283.17041), and were identified as rosmanol and epiisorosmanol. Compound **69** ([M-H]¯ at *m/z* 329.17580) was identified as carnosol based on its typical fragmentation pattern, starting with the loss of carbon dioxide (*m/z* 285.16604)^12,15^ and followed by the elimination of a methyl radical (*m/z* 270.16211). The same fragmentation pattern occurred in compound **80,** identified as 12-metoxy carnosic acid ([M-H]¯ at *m/z* 345.20721) with fragments of 301.21689 and 286.19385. Compound **78**, with a pseudomolecular ion at *m/z* 331.19153 [M-H]¯ was identified as carnosic acid owing to the presence of fragments corresponding to the loss of carbon dioxide and subsequent loss of an isopropyl radical (*m/z* 287.20175 and 244.14687). Compound **70** showed a precursor ion at [M-H]¯ at *m/z* 343.15524, which generated characteristic fragments *m/z* 315.16028 and *m/z* 299.160504 via the loss of ethylene and carbon dioxide, respectively. That allows us to identify compound **70** as rosmadial. Two pentacyclic triterpenoids were also detected in the tested extracts: compound **96** and **97**, which were tentatively identified as betulinic acid and ursolic acid, respectively, with quasimolecular ions at ([M-H]¯ at *m/z* 455.35340). The presence of these triterpenoids was also reported in *S. fruticosa* by Jash et al.^16^.

In the extracts studied, especially those with a high water content (SFH_2_O, SF30), a significant share of oligosaccharides and sugar acids in the total peak area of the identified compounds was also noted. Compounds **1**, **2** and **3** were identified tentatively as stachyose, raffinose and sucrose, as they are often major transport sugars in the *Salvia* species^31^. Compounds **4–8** were classified as sugar acids. The fragmentation pattern of compound **6** ([M-H]¯ at *m/z* 135.02875) was identical to that of l-threonic acid. Compound **8** ([M-H]¯ at *m/z* 149.0081) generated fragments *m/z* 72.99171, 59.01249 and 87.00734 that can be observed in negative ionization mode for l-( +)-tartaric acid.

Another major class of phytochemicals detected in *S. fruticosa* extracts was flavonoids. Most of the identified compounds belonging to this class have been assigned to flavones. Compound **24** was unambiguously identified as scutellarin by comparing the retention times, UV spectra and MS/MS fragmentation patterns with those of the commercial standard. Compounds **46** and **55** showed nearly the same precursor ions [M-H]¯ at *m/z* 299.0563 and 299.0562. Compound **46** produced most abundant fragments at *m/z* 284.03253 and 136.98682, similarly compound **55**. These data correspond with the fragmentation pattern of hispidulin or diosmetin. Since there was a difference in retention time, both compounds could be present in *S. fruticosa* extracts. Compound **49** showed a precursor ion at [M-H]¯ at *m/z* 285.04065 that formed specific product ions at *m/z* 133.02834, 151.00261, 175.03903, in line with these reported for luteolin by Velamuri et al.^17^. Compound **52** ([M-H]¯ at *m/z* 327.21786) was identified as salvigenin (pectolinarigenin-7-methyl ether) as this flavone has been reported previously in *S. fruticosa*. Compound **54** yielded the base peak [M-H]¯ at *m/z* 269.04578. Precursor ion and product ions at *m/z* 117.03332 and 151.00264 confirmed that this compound is apigenin. Compound **56** gave the precursor ion [M-H]¯ at *m/z* 329.0668, indicating that its molecular formula was C_17_H_14_O_7_. It produced prominent fragment ions at *m/z* 299.01981 attributable to the loss of two methyl groups, and 271.02472, owing to the further elimination of carbon monoxide. Therefore, this peak was identified as jaceosidin. Compound **60** was identified as cisimaritin based on a precursor ion [M-H]¯ at *m/z* 313.07190 and the diagnostic product ions at *m/z* 298.04694 and 283.02478, indicating the loss of two methyl radicals and 255.02974 from the elimination of carbon monoxide. Compound **62** ([M-H]¯ at *m/z* 283.06137) corresponds to an apigenin derivate considering the fragment at *m/z* 268.03772 and 117.03318. Characteristic fragment ion at *m/z* 240.04193 formed by the loss of carbon monoxide led to compound **62** being identified as genkwanin. Fragmentation patterns of apigenin, hispidulin, cirsimaritin and genkwanin were consistent with those reported by Koutsoulas et al.^12^. Compound **50** was the only type of flavonol aglycon detected in studied extracts. With the precursor ion [M-H]¯ at *m/z* 315.0513 and main MS/MS fragment at *m/z* 300.02756 resulting from the loss of methyl group this compound was identified as isorhamnetin. Compound **30** with a pseudomolecular ion [M-H]¯ at *m/z* 609.18329 did not show any fragmentation, but since it was previously reported in *S. fruticosa*^18,19^, it was tentatively identified as flavanone—hesperidin. Flavonoid glycosides found in this study were mainly glucosides with characteristic fragment of 162 Da, glucuronides (176 Da) and rutinosides (308 Da). Luteolin glucoside (compound **22** with [M-H]¯ at *m/z* 447.09344) is present in most publications concerning the chemical composition of *S. fruticosa* extracts^12,18–21^. Compound **26** showed a precursor ion [M-H]¯ at *m/z* 491.0836 and was identified as isorhamnetin glucuronide, reported earlier in *S. fruticosa* only by Gürbüz et al.^22^. Compound **27 (**[M-H]¯ at *m/z* 577.15668), identified as apigenin-rutinoside was also found in Greek sage by Cvetkovikj et al.^21^.

The presence of fatty acids was also observed in *S. fruticosa* extracts. Compounds **63** and **73** were identified tentatively as two polyunsaturated fatty acids. Compound **63** was assigned as dihydroxyoctadecadienoic acid (C_18_H_31_O_4_¯). Compound **73** produced precursor ion [M-H]¯ at *m/z* 295.22803 and characteristic fragments at *m*/*z* 277.21738 ([M-H-H_2_O]^−^ and 195.13837 [M-(CHO-(CH_2_)_4_-CH_3_)-H]¯, indicating the position of the hydroxyl group at 13 carbon atom. Thus, it was identified as 13-hydroxy-9,11-octadecadienoic acid. Also, in *S. fruticosa* extracts the presence of the glucoside of tuberonic acid (*m/z* 387.16644) (compound **14**) which is a growth hormone was observed.

### Quantitative analysis of major phytochemicals

A quantification of the main phenolic compounds in various extracts of *S. fruticosa* of the dry weight of plant material (mg/g DW) is presented in Table 1. The content of caffeic acid, scutellarin, salvianolic acid B, rosmarinic acid, carnosic acid and carnosol was calculated based on calibration curves of authentic standards, while the content of other compounds was estimated in relation to the most similar available standard.

**Table 1:** Content of major phenolic compounds (mg/g DW) determined in four different *S. fruticosa* extracts (SFH_2_O–water extract; SF30–30% ethanol extract; SF70–70% ethanol extract; SF100–ethanol) by HPLC–PDA.

Peak No.	Name	Content (mg/g DW)			
		SFH_2_O	SF30	SF70	SF100
16	Caffeic acid^1^	0.15 ± 0.01	0.15 ± 0.01	0.14 ± 0.01	0.13 ± 0.01
18	Hydroxyluteolin glucuronide^2^	1.86 ± 0.04	2.26 ± 0.25	2.61 ± 0.72	1.56 ± 0.6
19	Przewalskinic acid A^5^	2.38 ± 0.46^a^	1.45 ± 0.02^b^	1.47 ± 0.01^b^	1.45 ± 0.01^b^
21	Luteolin rutinoside^2^	1.65 ± 0.23^a^	1.81 ± 0.07^a^	1.7 ± 0.24^a^	1.03 ± 0.17^b^
22	Luteolin glucoside^2^	1.79 ± 0.04	1.98 ± 0.12	1.98 ± 0.39	1.69 ± 0.25
24	Scutellarin^1^	7.77 ± 0.48^a^	8.92 ± 1.56^a^	7.35 ± 0.9^a^	3.66 ± 0.36^b^
25	Isorhamnetin hexoside^4^	1.7 ± 0.03	1.71 ± 0.09	1.65 ± 0.25	1.46 ± 0.16
26	Isorhamnetin glucuronide^4^	1.99 ± 0.34	2.21 ± 0.17	2.03 ± 0.56	1.6 ± 0.37
37	Salvianolic acid B^1^	4.25 ± 0.65^b^	6.52 ± 0.48^a^	6.86 ± 0.93^a^	3.9 ± 0.63^b^
41	Rosmarinic acid^1^	4.96 ± 0.65^d^	25.27 ± 3.1^b^	31.56 ± 1.88^a^	12.85 ± 1.92^c^
44	Salvianolic acid K^5^	2.7 ± 0.53^b^	6.25 ± 1^a^	4.79 ± 0.7^a^	2.74 ± 0.83^b^
46	Diosmetin^2^	1.59 ± 0.04	1.58 ± 0.04	1.61 ± 0.04	1.53 ± 0.06
59	Rosmanol^6^	< LOQ	1.18 ± 0.1^b^	2.1 ± 0.25^a^	1.91 ± 0.1^a^
60	Cirsimaritin^3^	< LOQ	1.48 ± 0.01	1.53 ± 0.03	1.5 ± 0.02
61	Epirosmanol^6^	2.49 ± 0.06^b^	2.47 ± 0.14^b^	3.91 ± 0.59^a^	2.48 ± 0.28^b^
64	Epiisorosmanol^6^	< LOQ	3.65 ± 0.27^b^	6.43 ± 0.64^a^	5.65 ± 0.72^a^
69	Carnosol^1^	< LOQ	2.19 ± 0.15^c^	7.88 ± 1.33^a^	5.03 ± 0.44^b^
78	Carnosic acid^1^	1.9 ± 0.15^b^	2.13 ± 0.63^b^	13.88 ± 2.52^a^	14.82 ± 1.66^a^
80	Metoxy-carnosic acid^7^	< LOQ	< LOQ	4.12 ± 0.57	4.24 ± 0.76
89	Salviol^7^	1.77 ± 0.14^b^	< LOQ	7.37 ± 0.71^a^	6.42 ± 0.66^a^
	Total phenolic acids	14.44 ± 2.3^b^	39.64 ± 4.61^a^	44.82 ± 3.53^a^	21.07 ± 3.4^b^
	Total flavonoids	18.35 ± 1.2^ab^	21.95 ± 1.2^a^	20.46 ± 3.13^a^	14.03 ± 1.99^b^
	Total terpenoids	6.16 ± 0.35^b^	11.62 ± 1.29^b^	45.69 ± 6.61^a^	40.55 ± 4.62^a^

Concentration was calculated: ^1^based on authentic standards; ^2^as luteolin equivalent; ^3^as scutellarin equivalent; ^4^as quercetin equivalent; ^5^as salvianolic acid B equivalent; ^6^as carnosol equivalent; ^7^as carnosic acid equivalent; *LOQ* limit of quantification. The data are expressed as mean values of three independent experiments ± standard deviation. Values within a same line followed by the same letter are not significantly different (P > 0.05) (Tukey test). For identity of peaks, see Supplementary Table S1.

Overall, the most abundant compound in sage extracts is rosmarinic acid, which is a phenolic acid and a dimer of caffeic acid. The highest concentration of rosmarinic acid among all tested samples was found in SF70 (31.56 ± 1.88 mg/g DW), which is like the concentration of rosmarinic acid in *S. fruticosa* collected from Croatia (29.10 ± 0.21 mg/g DW), reported by Mervić et al.^23^. Even higher content (60.73 mg/g DW) was reported in methanolic extract from Greek variety of sage studied by Sarrou et al.^19^, but in this study the use of pure alcohol as a solvent did not result in the highest yield of rosmarinic acid. Rosmarinic acid concentration in the infusion (SFH_2_O) was much lower (4.96 ± 0.65 mg/g DW) than in other extracts which is not consistent with the findings of a similar comparison made for the Turkish variety of *S. fruticosa* by Tekin^18^. Caffeic acid was also detected in all studied extracts at similar concentrations (0.13–0.15 mg/g DW), which were ten times lower than those reported by Mervić et al.^23^. However, few salvianolic acids, which belong to major caffeic acid-derived trimers in sage plants, were present in greater amounts. The highest concentration of salvianolic acid B was obtained in SF70 and SF30 extracts (6.86 ± 0.93 mg/g DW and 6.52 ± 0.48 mg/g DW). Salvianolic acid K was the most abundant in ST30 extract with a concentration of 6.25 ± 1.0 mg/g DW. According to data presented by Cvetkovikj et al.^21^, the maximum concentration of salvianolic acid K among several studied Greek populations of *S. fruticosa* was 7.20 mg/g DW.

The most abundant terpenoid compounds in *S. fruticosa* are carnosic acid and carnosol, which both belong to the family of abietane diterpenoids^12^. The highest content of carnosic acid was observed in SF100 (14.82 ± 1.66 mg/g DW), followed by SF70 (13.88 ± 2.52 mg/g DW) which is not statistically different. These results are in line with the content measured in methanolic extract by Kallimanis et al.^24^ which was 12.5 ± 1.6 mg/g DW. The amount of carnosol in SF70 extract was 7.88 ± 1.33 mg/g DW and it was consistent with this reported by Sarrou et al.^19^. Salviol, the third most abundant terpenoid in studied extracts, is a meroterpenoid derived from abietane diterpenoid—ferruginol and is common in other Greek species of sage, e.g. *S. pomifera*^25^. This compound has not yet been reported in *S. fruticosa*; however it was present in most of studied extracts with the highest content: 7.37 ± 0.71 mg/g DW in SF70.

The third group of bioactives detected in *S. fruticosa* extracts were flavonoids. Scutellarin is one of the common flavonoids found in sage^26^. It was the most abundant flavonoid in the studied extracts with a similar yield: 7.77 ± 0.48 mg/g DW, 8.92 ± 1.56 mg/g DW and 7.35 ± 0.9 mg/g DW in SFH_2_O, SF30 and SF70 extracts, respectively. The concentrations of luteolin rutinoside and luteolin glucoside were similar in all studied extracts and ranged from 1.03 to 1.98 mg/g DW, which is not in line with data reported by Tekin et al.^18^, where the concentrations of these compounds in sage infusions were two to three times higher than in ethanol extracts.

Phenolic acids, flavonoids and terpenoids are typical bioactive compounds in *S. fruticosa*. As shown in Table 1, the extraction yield was highly affected by ethanol content in the solvent. The extraction with 70% ethanol provided the highest total yield of bioactives, in contrast to extraction with only water. The difference is clearly visible in the yield of phenolic acids and terpenoids, which in SF70 was three times higher and seven times higher, respectively. The maximum extraction yield of flavonoids was obtained with 30% ethanol, but it was only slightly higher than that obtained with 70% ethanol. Considering all groups of studied bioactives, 70% ethanol is concluded to be the best solvent among those tested for extraction of bioactive compounds from *S. fruticosa*.

### Antioxidant activity

The presence of compounds exhibiting antioxidant activity in the plant material has become an important aspect defining its health-promoting properties. In the case of various species of sage, their high antioxidant activity is caused mainly by phenolic compounds. In the presented studies, the total antioxidant activity was determined for *S. fruticosa* extracts prepared with extractants of different polarity. In addition, the antioxidant activity was determined for selected phenolic compounds typical for sage and belonging to various classes of secondary metabolites such as phenolic acids, flavones and diterpenoids.

**Figure 2:**
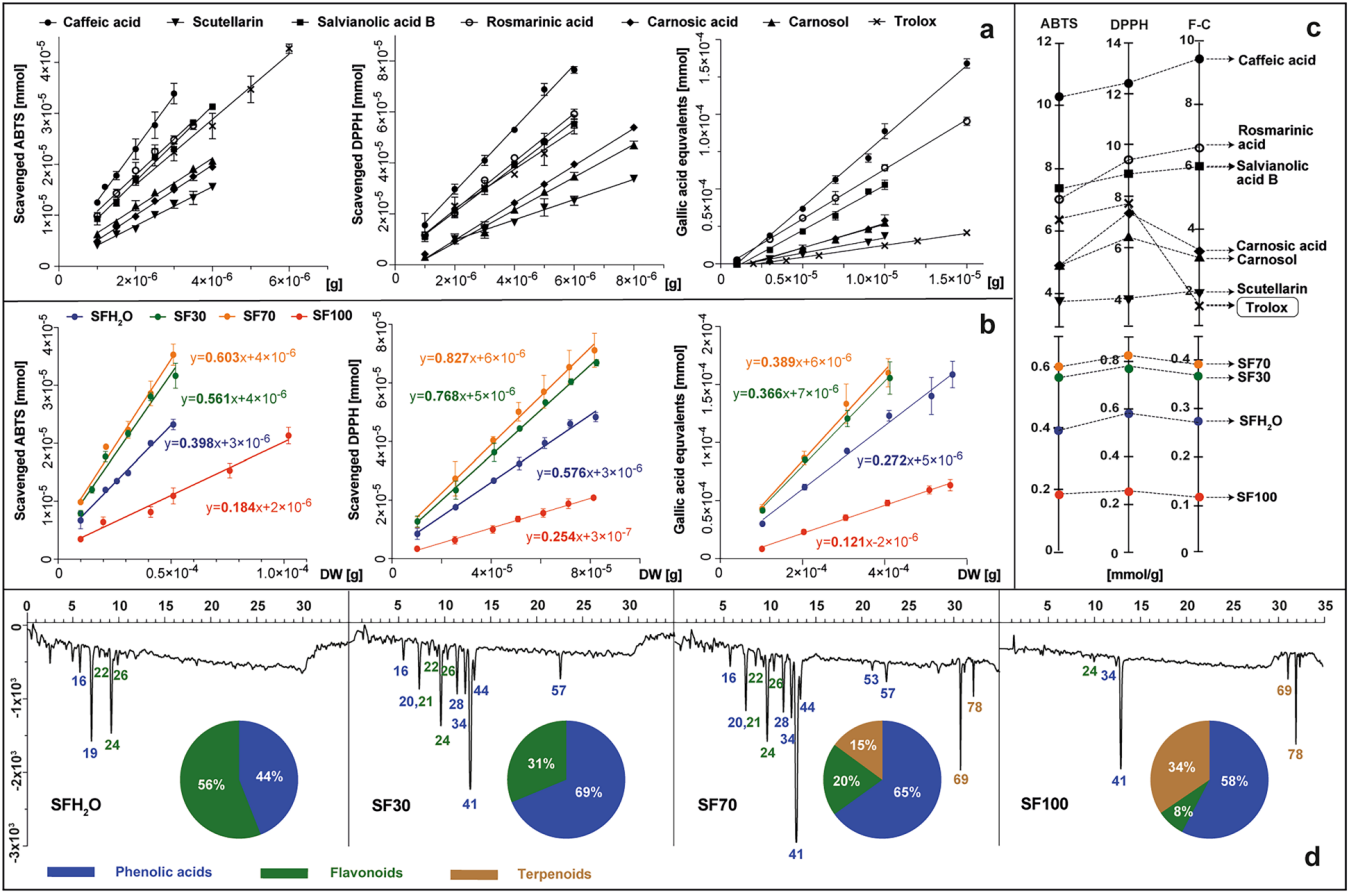
The antioxidant activity of standards (caffeic acid, scutellarin, salvianolic acid B, rosmarinic acid, carnosic acid, carnosol and trolox) and *S. fruticosa* extracts: SFH_2_O–water extract; SF30–30% ethanol extract; SF70–70% ethanol extract; SF100–ethanol extract, tested in vitro with ABTS, DPPH and F–C reagents presented as plots showing the dependency curves of reagent reduced by tested standards (**a**) or extracts (**b**) and expressed as slopes of the curves equal to the milomoles of reagent reduced by 1 g of tested sample (**c**) set with the antioxidant profiles of extracts, registered at 734 nm after post-column derivatization with ABTS, with the main classes of antioxidants on the pie charts (**d**). For the identity of peaks, see Supplementary Table S1.

The presented study compared the results of the three most popular spectrophotometric tests using ABTS, DPPH and Folin–Ciocalteu (F–C) reagents. ABTS and DPPH assays are used widely to determine free radical scavenging activity of extracts, as are pure compounds. For *S. fruticosa* extracts, the calculated antioxidant activity describes the number of ABTS or DPPH molecules reduced by antioxidants derived from 1 g of dried material after 10 min of reaction. These values were calculated in the linear range of the method and expressed as the slope of the line describing the relationship between the number of reduced millimoles of oxidants and various amounts of tested samples – as grams of dry matter in reaction mixtures (Fig. 2b).

The study also includes the method with the Folin–Ciocalteu reagent. It consists in the transfer of electrons in an alkaline environment from compounds with active hydroxyl groups to phosphomolybdic phosphotungstic acid complexes. The reducing capacity in this case was expressed as the number of millimoles of gallic acid equivalents, that formed blue complex and were derived from 1 g of dry matter of the plant. The same approach was used for the selected pure substances present in sage, such as: caffeic acid, carnosic acid, carnosol, salvianolic acid B, scutellarin, rosmarinic acid and additionally for the reference antioxidant—trolox (Fig. 2a). Such a method of determining and calculating the antioxidant activity of plant material and pure substances was described previously by Kusznierewicz et al.^27^ and Baranowska et al.^28^, respectively. The resulting slope values were plotted on separate axes for each test conducted for standards and samples (Fig. 2c). Each of the tested phenolic standard exhibited antioxidant activity, increasing inorder as follows: scutellarin < carnosol < carnosic acid < salvianolic acid B < rosmarinic acid < caffeic acid. Three of them – salvianolic acid B, rosmarinic acid and caffeic acid – were more efficient than trolox, a compound used commonly as reference in antioxidant activity determination assays. The antioxidant activity of all studied extracts of *S. fruticosa* was dose dependent in ABTS assay as well as in the DPPH test. Therefore, as the amount of extract added to the reaction mixture increases, so does the reducing power towards these radicals. The lowest total antioxidant activity was observed for the SF100 extract, followed by almost two times higher for SFH_2_O, and nearly four times higher for SF30 and SF70. The results of the F–C test followed the same trend as ABTS and DPPH with a Pearson correlation of 0.99, which indicates that the antioxidant activity of extracts depend greatly on the content of phenolics, as demonstrated by Lantzouraki et al.^29^.

Based on the contents of 6 phytochemicals selected for testing in the extracts (Table 1) and the antioxidant activities determined for them and for the extracts (Fig. 2a,c), we can determine the estimated contribution of these compounds to the total antioxidant activity of individual extracts. In the case of SFH_2_O, SF30 and SF70 extracts, 6 selected compounds, depending on the test, theoretically covered 21–30%, 45–63% and 64–86% of the determined total antioxidant activity, respectively. These results suggest the possible presence of other additional antioxidants in these extracts and/or their synergistic effects. Only in the case of the SF100 extract did the sum of the activities of the 6 standard compounds exceed the determined total activity of this extract ranging from 20 to 49%, depending on the test used. Such an observation may be the result of possible antagonistic interactions between the phytochemicals present in this kind of extract.

More detailed information on the types of antioxidants present in the tested *S. fruticosa* extracts was provided by using HPLC post-column derivatization with the ABTS reagent. The antioxidant profiles obtained by this method, as well as the contribution of different classes of antioxidants in the total antioxidant activity, are shown in Fig. 2d. In addition to the 6 standard antioxidants tested earlier, the *S. fruticosa* extracts also contained other antioxidants such as przewalskinic acid A, salviaflaside, luteolin rutinoside, luteolin glucoside, isorhamnetin glucuronide, coumaroyl caffeoylglycoside, salvianolic acid K and salvianolic acid F. Profiles characterized by the largest number and size of negative peaks indicating the reduction and discoloration of ABTS radicals were observed for the SF70 and SF30 extracts. Despite the similarity of the antioxidant profiles of these two samples the intensity of common signals was higher for SF70 and additional activity originating from diterpenoids was also noticed. Only in the profiles of SF70 and SF100 extracts were negative peaks originating from diterpenoids observed, with their share in the total antiradical activity at 15 and 34%, respectively. The main antioxidant in all the extracts containing ethanol was rosmarinic acid – the most abundant phenolic acid and one of the strongest antioxidants among studied standards. The same result was also reported for *S. officinalis* and *S. hispanica* extracts^30,31^.

In aqueous extract (SFH_2_O) the antioxidant activity originated mainly from two compounds: przewalskinic acid A and scutellarin, as rosmarinic acid extraction with water alone was less effective.

### Xanthine oxidase inhibitory activity

The enzyme xanthine oxidase (XO) catalyzes the oxidation of hypoxanthine and xanthine to uric acid, an excess of which in the blood causes gout to develop. During XO reoxidation, molecular oxygen acts as an electron acceptor, producing a superoxide radical and hydrogen peroxide. Consequently, XO is considered an important biological source of superoxide radicals which, together with other reactive oxygen species, contribute to the body’s oxidative stress and is involved in many pathological processes such as inflammation, atherosclerosis, cancer, ageing, etc.^32^. A recent therapeutic approach to hyperuricemia treatment is to inhibit the XO enzyme. Various drugs containing XO inhibitors (allopurinol, febuxostat) have been developed, the use of which is unfortunately associated with certain side effects. For this reason, there is a constant search for natural XO inhibitors that could provide an alternative to these synthetic compounds. There are some reports in the literature about the ability of several species of *Salvia* (*S. plebeia, S. miltiorrhiza, S. verbenaca*) to inhibit XO^33–35^, therefore, the possible occurrence of this activity was also tested in the studied *S. fruticosa* extracts. In addition, XO inhibitory activity was also determinedin selected phenolic compounds typical for sage such as: caffeic acid, carnosic acid, carnosol, salvianolic acid B, scutellarin, rosmarinic acid and additionally, for reference, the XO inhibitor allopurinol.

**Figure 3:**
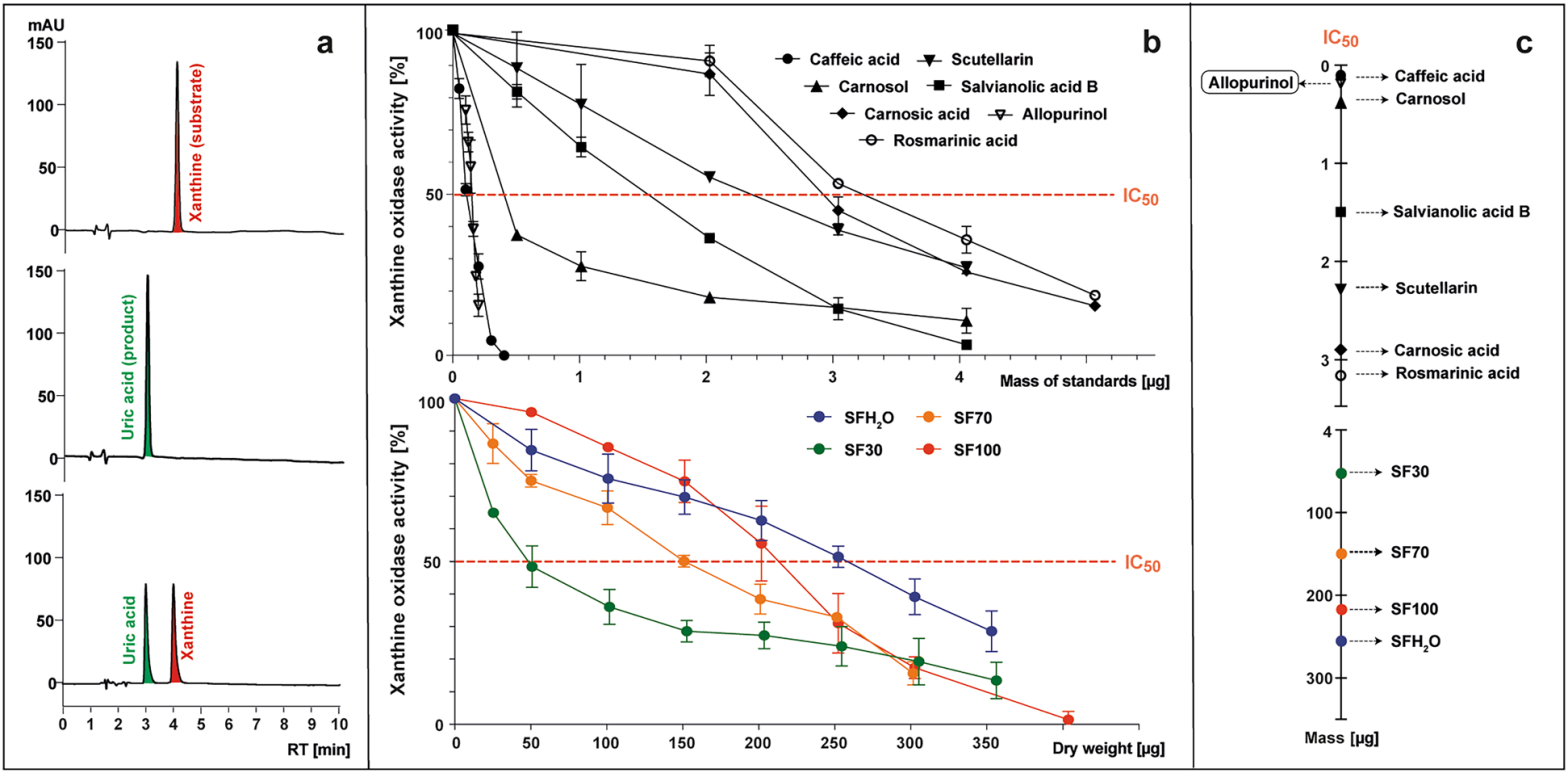
The examples of HPLC chromatograms at 285 nm of post-reaction mixtures containing (from top): xanthine; xanthine and xanthine oxidase (XO); xanthine, XO and inhibitor (**a**), which were the basis for preparing the plots representing the curves of XO activity in the presence of tested standards (caffeic acid, scutellarin, salvianolic acid B, rosmarinic acid, carnosic acid, carnosol and allopurinol) or *S. fruticosa* extracts (SFH_2_O–water extract; SF30–30% ethanol extract; SF70–70% ethanol extract; SF100 – ethanol extract) (**b**), which were used to determine the parameter IC_50,_ meaning the micrograms of tested sample needed to reduce XO activity to 50% (**c**).

The transformation of xanthine (substrate) to uric acid (product) by XO with or without the presence of tested samples was monitored with the use of HPLC-PAD at 285 nm (Fig. 3a). The enzyme activity was calculated as the percentage of the uric acid peak area formed in the presence of the tested sample compared to the control without the addition of the sample (Fig. 3b). The inhibition of the XO enzyme was expressed as an IC_50_ value, meaning the mass of standard or dry weight of sample (μg) capable of reducing enzyme activity to 50% (Fig. 3b,c).

The known XO inhibitor allopurinol was used as a reference, with an IC_50_ value of 0.15 μg (5.5 µM). All the studied standards showed the XO inhibitory activity with an IC_50_ ranging from 0.1 to 3.15 μg (2.8–43.8 µM). XO inhibitory activity increased in order as follows: rosmarinic acid < carnosic acid < scutellarin < salvianolic acid B < carnosol < caffeic acid. Caffeic acid showed the lowest IC_50_ value (0.1 μg; 2.8 µM), indicating the strongest XO inhibitory activity among the tested compounds. It was even stronger than allopurinol, which is inconsistent with the data presented by Wan et al.^36^ and Flemmig et al.^37^, where the IC_50_ of caffeic acid was almost 8 or 2 times lower than that of allopurinol, respectively. These differences may result from the origin of the XO selected for the tests. In the cited studies, an oxidase from bovine milk was used, while for this study a microbial oxidase was selected. In this study, rosmarinic acid had the lowest to inhibit XO (3.2 μg; 43.8 µM), but Ghallab et al.^38^ reported that synergistic combination of allopurinol and rosmarinic acid can lower the dosage of synthetic drugs needed. XO was inhibited by all studied *S. fruticosa* extracts, although over 1000 times less effectively than by allopurinol, in line with data reported for other *Salvia* species. Scutellarin and other flavones have been described previously as strong inhibitors of XO^39^. Despite only a slight difference of total flavonoids content between SF30 and SF70 extract, and the contents of other anti-inflammatory compounds being more favorable for the SF70 extract, SF30 had the strongest ability to inhibit the XO activity. The IC_50_ value for SF30 was 50 μg and based on this parameter, the potential anti-inflammatory activity of SF70, SF100 and SFH_2_O extracts was determined as 3, 4 and 5 times weaker, respectively.

## Conclusions

In this study, four extracts of *S. fruticosa* were characterized and compared in terms of chemical composition and bioactivity. The proposed UPLC-MS/MS analysis led to the identification of 95 compounds in total. This study presents the precise composition of *S. fruticosa* extract obtained with boiling water, which is a traditional form of sage preparation, that presents the benefits of alternative extraction methods. The greatest diversity and abundance of secondary metabolites was obtained in an extract prepared with 70% ethanol. Considering all the obtained results, it can be concluded that the extraction of *S. fruticosa* with the aid of combined ethanol and water provides the best results in terms of tested bioactivities. Additionally, testing several standards in parallel with the analysis of extracts allowed not only quantitative analysis, but also helped to indicate the role of these compounds in bioactivities shown by the extracts. Caffeic acid, scutellarin, salvianolic acid B, rosmarinic acid, carnosic acid and carnosol are present in many other plant species, so these data can be useful in a wide range of research topics concerning medicinal plants. All the tested extracts exhibited antioxidant activity in a dose-dependent manner and the strongest was the extract prepared with 70% ethanol (SF70). On the other hand, it was the 30% ethanolic extract (SF30) that showed the strongest anti-inflammatory effect by inhibiting XO. The proposed method of determining the anti-inflammatory activity by HPLC allowed us to examine both individual compounds and complicated mixtures such as herbal extracts. The target peak of uric acid was well separated from the components of the extracts. Stopping the reaction with HCl allowed us to perform the batch HPLC analysis on many samples, without the risk of unwanted changes owing to the measurement delay.

## Materials and methods

### Plant material

The plant material comprised dried Greek sage leaves, known as *Salvia fruticosa* Mill. 1768 or *Salvia triloba* L.f. 1782. It was acquired from an ecological producer (GRECO Bio Products). According to supplier’s information, the plant material was air-dried traditionally, without exposure to sunlight.

### Chemicals and reagents

The reagents of analytical, HPLC or MS grade acetonitrile, ethanol, methanol, formic acid, hydrochloric acid, sodium hydroxide and reagents for antioxidant activity determination, including 2,2′-azinobis (3-ethylbenzothiazoline-6-sulfonic acid) diammonium salt (ABTS), 2,2-diphenyl-1-picrylhydrazyl (DPPH), Folin–Ciocalteu’s phenol reagent (FC), trolox and gallic acid, as well as the reagents for anti-inflammatory activity determination – allopurinol, uric acid, xanthine and microbial XO 10 U/mg – were purchased from Sigma Aldrich (St. Louis, MO, USA). The phosphate buffer was prepared with Na_2_HPO_4_ × 2H_2_O and NaH_2_PO_4_ × 2H_2_O (Chempur, Poland). Standards of caffeic acid, carnosol, salvianolic acid B, scutellarin, luteolin, quercetin, rosmarinic acid and carnosic acid were purchased from Extrasynthese (France).

### Methods

All the methods were carried out in accordance with relevant Institutional guidelines and regulations.

#### Standard solutions preparation

Stock standard solutions at a concentration of 1 mg/mL were prepared using the appropriate solvent. Methanol was used to dissolve carnosic acid, rosmarinic acid, carnosol, scutellarin and caffeic acid, and methanol/water (70:30, v/v) was used to dissolve salvianolic acid B. Working standard solutions were prepared in triplicate by diluting the stock solution of each standard in a range of concentrations appropriate to the linear response of each method.

#### Sample preparation

UAE was performed with the aid of ethanol and water in various proportions for a comparative study. The extractants used for this study were: water (SFH_2_O), ethanol/water (30:70, v/v) (SF30), ethanol/water (70:30, v/v) (SF70) or ethanol (SF100). The 100 mg of ground plant material was extracted with 2 mL of extractant. The extraction was assisted by ultrasound (10 min, 30 °C). Then the samples were centrifuged at 13,000 rpm for 5 min and supernatants were collected. The extracts were stored at − 20 °C until use. Each extraction was conducted in triplicate.

#### Metabolomic profiling using LC-Q-Orbitrap HRMS

The Ultimate 3000 UHPLC system (Thermo Scientific Dionex), equipped with a quaternary.

pump, column compartments, autosampler and a PDA detector controlled by Chromeleon 7.2.8 software (Thermo Fisher Scientific,Waltham, MA, USA, and Dionex Softron GmbH Part of Thermo Fisher Scientific, Bremen, Germany), coupled with a high-resolution Thermo.

Q-Exactive™ Focus quadrupole-Orbitrap mass spectrometer (Thermo, Bremen, Germany) were employed for analysis. Chromatography separations were performed using a Kinetex® column (150 × 4.6 mm, 5 µm, Phenomenex). A linear gradient was obtained with 0.1% formic acid in water (solvent A) and 0.1% formic acid in acetonitrile (solvent B), with a flow rate of 0.8 mL/min. Composition of solvents changed from 15% of solvent B to 40% in 25 min, then increased linearly to 100% up to 30 min and kept at this level up to 35 min of analysis. The injection volume was 2 μL.

The HESI parameters in negative ion acquisition mode were as follows: sheath gas flow rate, 35 arb; auxiliary gas flow rate, 15 arb; sweep gas flow rate, 3 arb; spray voltage, 2.5 kV; capillary temperature, 350 °C; S-lens RF level, 50; and heater temperature, 300 °C. The full MS scan was set as follows: resolution, 70,000 FWHM; AGC target, 2e^5^; max inject time, 100 ms; and scan range, 120–1200 m*/z*. The data-dependent MS^2^ parameters were as follows: resolution, 17,500 FWHM; isolation window, 3.0 m*/z*; collision energy, 30 eV; AGC target, 2e^5^; max inject time, 100 ms. The external mass calibration and the quadrupole calibration were carried out daily. For the calibration, a mixture containing n-butylamine, caffeine, Met-Arg-Phe-Ala (MRFA), and Ultramark 1621 was used. All samples were run in triplicate followed by injecting the blank H_2_O/EtOH (1:1, v/v). Data were analyzed using Compound Discoverer 2.1 software.

For quantitative determination of major phytochemicals – caffeic acid, carnosic acid, carnosol, luteolin, quercetin, rosmarinic acid, salvianolic acid B and scutellarin – the calibration curves were generated on peak areas obtained with chromatographic analysis of the serial dilutions of authentic standards.

#### Antioxidant profiling by post-column derivatization with ABTS

Antioxidant profiles were obtained for *S. fruticosa* extracts using an HPLC-PAD system (1200 series, Agilent Technologies, Wilmington, DE, USA) coupled with a Pinnacle PCX Derivatization Instrument (Pickering Laboratories Inc., Mountain View, California, USA.) and a UV–Vis detector (Agilent Technologies, Wilmington, DE, USA). The chromatographic column and conditions of chromatographic separation were the same as in the case of LC-HRMS analysis. The post-column derivatization with ABTS reagent was carried out according to the methodology reported by Kusznierewicz et al.^40,41^ with slight modification. Stream of eluate leaving the column was mixed with a stream of methanolic ABTS solution (0.2 mL/min) and directed to the reaction loop (1 mL, 130 °C). The reduction of the ABTS radical by extract components was monitored at 734 nm. The contribution of the separated analyte to the total antioxidant activity of extracts was estimated on the assumption that 100% is represented by the sum of the negative peak areas integrated in chromatograms obtained after derivatization with ABTS.

#### Total antioxidant activity determination

The antioxidant activity of – caffeic acid, carnosic acid, carnosol, rosmarinic acid, salvianolic acid B, scutellarin and *S. fruticosa* extracts – was determined by spectrophotometric tests using ABTS, DPPH and Folin–Ciocalteu (F–C) reagents. The procedure and calculations were based on method described by Kusznierewicz et al.^27^. For tests with radicals, the data were used to generate a plot of linear dependence between the different content of tested standards or *S. fruticosa* samples calculated as gram of dry weight present in the reaction mixtures and the number of scavenged millimoles of ABTS or DPPH radical. The slopes of the obtained straight lines were then used for expressing the total antioxidant activity as the mean of ABTS or DPPH millimoles, reduced by 1 g of tested sample. For the F–C test the results were calculated using the calibration curve of gallic acid. The antioxidant activity of the tested samples was expressed as a slope of the line that represents the relationship between the mass of standards or plant material [g] and amount of gallic acid equivalents [mmol] present in reaction mixtures after 60 min.

#### Xanthine oxidase inhibitory activity

The ability of – caffeic acid, carnosic acid, carnosol, rosmarinic acid, salvianolic acid B and scutellarin, as well as *S. fruticosa* extracts – to inhibit XO was determined. The stock solutions of xanthine (substrate) and uric acid (product) (1.5 mM) were prepared in an NaOH aqueous solution (1 M). The working solutions of these compounds (150 μM) were prepared by dilution of stock solutions with a phosphate buffer (10 mM, pH 7.7). An enzyme solution with an activity of approximately 0.5 U/mL was prepared in the same phosphate buffer. The same buffer was also used to dilute stock solutions of phytochemical standards and *S. fruticosa* extracts. The diluted samples with different concentrations of standard or plant extract (85 µL) were mixed with an XO solution (30 µL) and preincubated for 15 min at 25 °C, followed by the addition of 60 µL of a 150 µM xanthine solution (substrate). The mixtures were incubated for 60 min at 25 °C, then 25 µL of HCl solution (1 M) was added to stop the enzymatic reaction.

The samples were analyzed using an HPLC-PAD system (1200 series, Agilent Technologies, Wilmington, DE, USA) equipped with a Kinetex® column (150 × 4.6 mm, 5 µm, Phenomenex). A two-component mobile phase was used, in which component A was water acidified with formic acid (1%) and component B was methanol. The mobile phase was pumped at flow rate of 1 mL/min according to the gradient elution program: 0 min – 100% A, 8 min – 18% B, 12 min – 80% B, 18 min – 100% B and finally, the initial conditions were held for 5 min as a re-equilibration step. The injection volume was 30 μL. The inhibition of the XO enzyme was expressed as an IC_50_ value, meaning the mass of standard or dry weight of the sample (μg) that could reduce enzyme activity to 50%. Enzyme activity was calculated as the percentage of the uric acid peak area (RT 3.1 min) that was formed in the presence of extracts compared to the control sample without the addition of extracts.

### Statistical analysis

All data were analyzed using Microsoft Excel and GraphPad Prism 8 software. Each experiment was carried out in triplicate (n = 3) and data were presented as mean ± standard deviation (SD). The n values representing antioxidant activity were calculated using linear regression analysis. The degree of association between the two variables was determined by calculating Pearson’s correlation coefficient. The differences between the results were determined by the ordinary one-way ANOVA and post hoc Tukey’s multiple comparisons test. All values where P < 0.05 were considered statistically significant.

### Supplementary Information

Supplementary Table S1.

## Data Availability

The datasets analyzed during the current study are available from the corresponding author on reasonable request.
