# Supplementary material for: Phytochemical screening and biological evaluation of Greek sage (Salvia fruticosa Mill.) extracts
Source: Sci Rep. 2023 Dec 15;13:22309. doi: 10.1038/s41598-023-49695-w (PMC10724190; doi:10.1038/s41598-023-49695-w)
Supplement: Supplementary file 1 — Supplementary Table S1. [file 41598_2023_49695_MOESM1_ESM.docx]

**Supplementary Table S1** Major compounds identified by LC-Q-Orbitrap HRMS in different extracts of Greek sage (*Salvia fruticosa*).

| No. | Compound | RT [min] | Formula | Expected mass [Da] | Experimental mass [Da] | Δmass [ppm] | Adduct type | Adduct mass m/z | MS/MS | Class | IDL |
| --- | --- | --- | --- | --- | --- | --- | --- | --- | --- | --- | --- |
| 1 | Stachyose | 1.85 | C_24_H_42_O_21_ | 666.22187 | 666.2222 | -0.5 | [M+FA-H] ^−^ | 711.22083 | 383.11978; 179.05524; 341.10089; 485.15118 | OS | [3] |
| 2 | Raffinose | 1.87 | C_18_H_32_O_16_ | 504.16904 | 504.16923 | -0.4 | [M+FA-H] ^−^ | 549.16766 | 89.02304; 113.02319; 179.05521; 160.06041 | OS | [3] |
| 3 | Sucrose | 1.89 | C_12_H_22_O_11_ | 342.11622 | 342.11633 | -0.3 | [M-H] ^−^ | 341.10901 | 59.01249; 89.02302; 101.02306; 113.02313; 179.05522 | OS | [3] |
| 4 | Gluconic acid | 1.92 | C_6_H_12_O_7_ | 196.05831 | 196.05763 | 3.4 | [M-H] ^−^ | 195.05035 | 75.00736; 59.01249; 87.00737; 71.01247; 105.01803 | SA | [2] |
| 5 | Xylonic acid | 1.94 | C_5_H_10_O_6_ | 166.04774 | 166.04683 | 5.5 | [M-H] ^−^ | 165.03952 | 75.00735; 59.01250; 72.99172; 87.00739 | SA | [3] |
| 6 | Threonic acid | 1.96 | C_4_H_8_O_5_ | 136.03718 | 136.03604 | 8.3 | [M-H]^−^ | 135.02875 | 75.00736; 59.01250; 72.99171; 71.01246 | SA | [3] |
| 7 | Heptose | 1.96 | C_7_H_14_O_7_ | 210.074 | 210.07339 | -2.7 | [M-H] ^−^ | 209.06613 | 71.01244; 60.01577; 85.02808; 115.00249 | SA | [3] |
| 8 | Tartaric acid | 2.02 | C_4_H_6_O_6_ | 150.05283 | 150.01543 | -6.7 | [M-H] ^−^ | 149.00810 | 72.99171; 59.01249; 87.00734; 75.00732; 110.79290 | SA | [3] |
| 9 | 3-Dehydro-L-threonic acid | 2.08 | C_4_H_6_O_5_ | 134.02153 | 134.02039 | 8.5 | [M-H] ^−^ | 133.01309 | 71.01244; 72.99170; 59.01247; 115.00237 | SA | [3] |
| 10 | Citric acid | 2.16 | C_6_H_8_O_7_ | 192.02701 | 192.02630 | 3.7 | [M-H] ^−^ | 191.01912 | 87.00738; 111.00752; 67.01758; 57.03324; 85.02813 | CA | [3] |
| 11 | Trans-Caffeic acid [apiosyl-(1->6)-glucosyl] ester | 3.11 | C_20_H_26_O_13_ | 474.13730 | 474.13758 | 0.5 | [M-H] ^−^ | 473.13030 | 161.02345; 179.03410; 89.02302; 162.02689 | PA | [3] |
| 12 | Chlorogenic acid | 4.14 | C_16_H_18_O_9_ | 354.09509 | 354.09536 | -0.8 | [M-H] ^−^ | 353.08790 | 191.05534; 173.04457; 85.02807; 135.04413; 292.56693 | PA | [2] |
| 13 | Salicylic acid | 4.71 | C_7_H_6_O_3_ | 138.03170 | 138.03058 | 8.1 | [M-H] ^−^ | 137.02328 | 108.02039; 136.01541; 91.01755; 81.03314; 65.001195 | CA | [2] |
| 14 | Tuberonic acid glucoside | 4.72 | C_18_H_28_O_9_ | 388.17334 | 388.17357 | -0.6 | [M-H] ^−^ | 387.16644 | 59.01249; 89.02304; 101.02305; 163.11189; 207.10207 | JAS-Gly | [3] |
| 15 | Daphnetin | 5.42 | C_9_H_6_O_4_ | 178.02661 | 178.02679 | -1.0 | [M-H] ^−^ | 355.04633 | 311.05634; 283.06125; 175.03923; 134.03616; 293.04575; 193.04990 | HCU | [2] |
| 16 | Caffeic acid | 5.59 | C_9_H_8_O_4_ | 180.04226 | 180.04146 | 4.4 | [M-H] ^−^ | 179.03419 | 135.04414; 134.03619; 89.03828; 117.03338; 107.04903; 162.49875 | PA | [1] |
| 17 | Unknown | 6.47 | C_17_H_30_O_10_ | 394.18390 | 394.18416 | 0.7 | [M+FA-H] ^−^ | 439.18222 | 89.02301; 59.01248; 71.01245; 101.02304; 113.02306; 149.04466 | [-] | [-] |
| 18 | 6‑Hydroxyluteolin ‑7‑O‑glucuronide | 7.35 | C_21_H_18_O_13_ | 478.07475 | 478.07496 | -0.4 | [M-H] ^−^ | 477.06769 | 301.03549; 113.02310; 302.03888; 303.04080; 343.04648 | FON-Gly | [2] |
| 19 | Przewalskinic acid A | 7.52 | C_18_H_14_O_8_ | 358.06887 | 358.06908 | -0.6 | [M-H] ^−^ | 357.06183 | 109.02821; 159.04413; 269.08185; 175.03928; 135.04410; 203.03433 | PA | [2] |
| 20 | Salviaflaside isomer | 8.37 | C_24_H_26_O_13_ | 522.13734 | 522.13755 | 0.4 | [M-H] ^−^ | 521.13012 | 161.02344; 179.03412; 135.04398; 197.04491; 359.07669 | PA | [2] |
| 21 | Luteolin rutinoside | 8.39 | C_27_H_30_O_15_ | 594.15848 | 594.15891 | -0.7 | [M-H] ^−^ | 593.15170 | 285.04047; 286.04370; 352.39798; 97.99312; 113.61324 | FON-Gly | [2] |
| 22 | Luteolin‑7‑glucoside | 9.25 | C_21_H_20_O_11_ | 448.10057 | 448.10082 | -0.6 | [M-H] ^−^ | 447.09344 | 285.04034; 286.04373; 284.03268; 327.05099; 133.02786 | FON-Gly | [2] |
| 23 | Unknown glycoside | 9.30 | C_18_H_26_O_8_ | 370.16277 | 370.16296 | -0.5 | [M-H] ^−^ | 369.15555 | 165.09119; 59.01250; 101.02307; 71.01247; 85.02811; 113.02323 | [-] | [-] |
| 24 | Scutellarin | 9.64 | C_21_H_18_O_12_ | 462.07983 | 462.08011 | -0.6 | [M-H] ^−^ | 461.07288 | 285.04041; 286.04395; 113.02309; 85.02813; 327.05154; 357.06161 | FON | [1] |
| 25 | Isorhamnetin O-hexoside | 9.92 | C_22_H_22_O_12_ | 478.11113 | 478.11130 | -0.4 | [M-H] ^−^ | 477.10416 | 315.05124; 299.01996; 300.02713; 316.05460; 462.08109; 285.01019 | FOL-Gly | [2] |
| 26 | Isorhamnetin glucuronide | 10.32 | C_22_H_20_O_13_ | 492.09040 | 492.09082 | -0.9 | [M-H] ^−^ | 491.08360 | 315.05136; 300.02774; 316.05466; 301.03116; 113.02317; 85.02813 | FOL-Gly | [2] |
| 27 | Apigenin-rutinoside | 10.42 | C_27_H_30_O_14_ | 578.16356 | 578.16401 | -0.8 | [M-H] ^−^ | 577.15668 | 269.04559; 270.04904; 135.04398; 179.03415; 311.05673 | FON-Gly | [2] |
| 28 | Coumaroyl caffeoylglycoside | 11.12 | C24H24O11 | 488.13186 | 488.13222 | 0.7 | [M-H] ^−^ | 487.12494 | 161.02341; 323.07721; 163.03905; 179.03404 | PA | [3] |
| 29 | Hispidulin-rutinoside | 11.31 | C_28_H_32_O_15_ | 608.17413 | 608.17456 | -0.7 | [M-H] ^−^ | 607.16742 | 299.05627; 284.03229; 301.06915; 341.06674 | FON-Gly | [2] |
| 30 | Hesperidin | 11.33 | C_28_H_34_O_15_ | 610.18978 | 610.19019 | -0.7 | [M-H] ^−^ | 609.18329 | - | FVA | [2] |
| 31 | Dihydrojasmonic acid glucoside | 11.34 | C_18_H_30_O_8_ | 374.19407 | 374.19422 | -0.4 | [M-H] ^−^ | 373.18680 | 59.01247; 211.13344; 89.02300; 101.02300; 71.01247; 193.12256; | JAS-Gly | [3] |
| 32 | Kaempferol-7-O-glucoside | 11.70 | C_21_H_20_O_11_ | 448.10057 | 448.10105 | -1.1 | [M-H] ^−^ | 447.09357 | 285.0450; 286.04385; 135.04352; 96.95869 | FON-Gly | [2] |
| 33 | Azelaic acid | 11.83 | C_9_H_16_O_4_ | 188.10486 | 188.10420 | 3.5 | [M-H] ^−^ | 187.09683 | 97.06452; 123.08032; 125.09541; 57.03325; 95.04876;167.12332 | CA | [3] |
| 34 | Salviaflaside  isomer | 12.07 | C_24_H_26_O_13_ | 522.13734 | 522.13771 | 0.71 | [M-H] ^−^ | 521.13043 | 323.07733; 161.02341; 179.03412; 221.04514; 135.04395 | PA | [2] |
| 35 | Hispidulin 7-glucoside | 12.15 | C_22_H_22_O_11_ | 462.11622 | 462.11660 | -0.8 | [M-H] ^−^ | 461.10941 | 283.02484; 284.02975; 298.04767; 299.05493; 297.04062; 446.08615 | FON-Gly | [2] |
| 36 | Apigenin glucuronide | 12.22 | C_21_H_18_O_11_ | 446.08492 | 446.08517 | -0.6 | [M-H] ^−^ | 445.07780 | 269.04559; 270.04898; 113.02312; 85.02811; 59.01249; 99.00742 | FON-Gly | [2] |
| 37 | Salvianolic acid B | 12.22 | C_36_H_30_O_16_ | 718.15339 | 718.15392 | -0.7 | [M-H] ^−^ | 717.14661 | 295.06125; 493.11429; 519.09338; 321.04037; 339.05090; 537.10413 | PA | [1] |
| 38 | Luteolin O-malonyl hexoside | 12.29 | C_24_H_22_O_14_ | 534.10096 | 534.10127 | -0.6 | [M-H] ^−^ | 533.09387 | 489.10413; 285.04044; 490.10754; 284.03265; 447.09302; 327.05182 | FON-Gly | [2] |
| 39 | Isosalvianolic acid B | 12.35 | C_36_H_30_O_16_ | 718.15339 | 718.15382 | -0.6 | [M-H] ^−^ | 717.14667 | 295.06125; 493.11429; 519.09357; 321.04044; 339.05112; 537.10425 | PA | [2] |
| 40 | Sagerinic acid | 12.76 | C_36_H_32_O_16_ | 720.16904 | 720.16946 | -0.6 | [M-H] ^−^ | 719.16210 | 359.07727; 161.02338; 197.04482; 360.08304; 179.03404; 135.04358 | PA | [2] |
| 41 | Rosmarinic acid | 12.77 | C_18_H_16_O_8_ | 360.08452 | 360.08464 | -0.3 | [M-H] ^−^ | 359.07730 | 161.02339; 72.99169; 179.03407; 135.04396; 197.04486; 123.04386 | PA | [1] |
| 42 | Rosmarinic acid derivative | 12.79 | C_18_H_17_ClO_8_ | 396.06339 | 396.06123 | 0.1 | [M-H] ^−^ | 395.05392 | 161.2345; 197.04491; 179.03413; 72.99171; 135.04399; 198.04863 | PA | [3] |
| 43 | Hispidulin glucuronide | 12.81 | C_22_H_20_O_12_ | 476.09548 | 476.09553 | -0.1 | [M-H] ^−^ | 475.08835 | 299.05615; 284.03256; 113.02310; 85.02808; 300.05948; 285.03256 | FON-Gly | [2] |
| 44 | Salvianolic acid K | 13.17 | C_27_H_24_O_13_ | 556.12170 | 556.12203 | -0.6 | [M-H] ^−^ | 555.11469 | 161.02342; 135.04396; 197.04488; 359.07721; 179.03409; 313.07031 | PA | [2] |
| 45 | Luteolin 7-O-glucuronide | 13.27 | C_21_H_18_O_12_ | 462.07983 | 462.08027 | -0.9 | [M-H] ^−^ | 461.07280 | 285.04037; 286.04388; 113.02315; 133.02884 | FON-Gly | [2] |
| 46 | Diosmetin/Hispidulin | 15.01 | C_16_H_12_O_6_ | 300.06339 | 300.06358 | -0.6 | [M-H] ^−^ | 299.05620 | 284.03253; 136.98682; 165.98978; 109.99957; 200.04723; 65.00191 | FON | [2] |
| 47 | Hydramacroside A | 15.81 | C_28_H_36_O_12_ | 564.22068 | 564.22105 | -0.7 | [M-H] ^−^ | 563.21370 | 387.16623; 175.03917; 193.05005; 207.10223; 59.01235; 531.18658 | SI | [3] |
| 48 | Methyl rosmarinate | 16.42 | C_20_H_28_O_5_ | 348.19368 | 348.19388 | -0.6 | [M-H] ^−^ | 347.18655 | 283.17038; 284.17365; 301.18063; 329.17606; 227.10715; 243.10255 | PA | [2] |
| 49 | Luteolin | 17.59 | C_15_H_10_O_6_ | 286.04774 | 286.04792 | -0.6 | [M-H] ^−^ | 285.04065 | 133.02834; 151.00261; 107.01257; 175.03903; 149.02339; 199.03967 | FON | [2] |
| 50 | Isorhamnetin | 17.75 | C_16_H_12_O_7_ | 316.05831 | 316.05860 | -0.9 | [M-H] ^−^ | 315.05130 | 300.02756; 136.98685; 228.04213; 65.00186; 200.04726; 216.04251 | FOL | [2] |
| 51 | Ethyl caffeate | 17.76 | C_11_H_12_O_4_ | 208.07356 | 208.07305 | 2.4 | [M-H] ^−^ | 207.06577 | 133.02834; 135.04405; 134.03676; 161.02338; 179.03391; 106.04146 | PA | [3] |
| 52 | Salvigenin | 20.72 | C_18_H_16_O_6_ | 328.22498 | 328.22517 | -0.6 | [M-H] ^−^ | 327.21786 | 211.13354; 229.14413; 171.10185; 183.13815; 97.06453; 85.02822 | FON | [2] |
| 53 | Salvianolic acid F isomer | 20.87 | C_17_H_14_O_6_ | 314.07904 | 314.07916 | -0.4 | [M-H] ^−^ | 313.07202 | 161.02347; 151.03905; 133.02827; 133.02827; 123.04395; 208.59291 | FON | [2] |
| 54 | Apigenin | 21.45 | C_15_H_10_O_5_ | 270.05283 | 270.05305 | -0.8 | [M-H] ^−^ | 269.04578 | 117.03332; 151.00264; 149.02344; 107.01257; 65.00194; 159.04424 | FON | [2] |
| 55 | Diosmetin/Hispidulin | 21.77 | C_16_H_12_O_6_ | 300.06339 | 300.06358 | -0.6 | [M-H] ^−^ | 299.05630 | 284.03265; 136.98689; 285.03595; 65.00193; 212.04745; 200.04720 | FON | [2] |
| 56 | Jaceosidin | 22.42 | C_17_H_14_O_7_ | 330.07396 | 330.07410 | -0.4 | [M-H] ^−^ | 329.06680 | 299.01981; 313.03537; 271.02472; 285.04028; 199.03938; 133.02777 | FON | [2] |
| 57 | Salvianolic acid F isomer | 22.49 | C_17_H_14_O_6_ | 314.07904 | 314.07922 | -0.6 | [M-H] ^−^ | 313.07205 | 161.02342; 133.02831; 162.02675; 151.03902; 123.04391; 134.03168 | PA | [2] |
| 58 | Salvianolic acid C | 23.14 | C_26_H_20_O_10_ | 492.10565 | 492.10599 | -0.7 | [M-H] ^−^ | 491.09863 | 179.03409; 267.06619; 161.02341; 311.05658; 283.06091; 150.02341 | PA | [2] |
| 59 | Rosmanol | 26.35 | C_20_H_26_O_5_ | 346.17803 | 346.17811 | -0.2 | [M-H] ^−^ | 345.17075 | 301.18097; 283.17038; 302.18439; 284.17361; 258.12570; 227.10713 | DT | [2] |
| 60 | Cirsimaritin | 27.03 | C_17_H_14_O_6_ | 314.07904 | 314.07931 | -0.9 | [M-H] ^−^ | 313.07190 | 283.02478; 284.02805; 255.02974; 297.04031; 298.04694; 163.00259 | FON | [2] |
| 61 | Epirosmanol | 28.16 | C_20_H_26_O_5_ | 346.17803 | 346.17810 | -0.2 | [M-H] ^−^ | 345.17070 | 283.17038; 284.17371; 268.14685; 227.10704; 301.18076 | DT | [2] |
| 62 | Genkwanin | 29.24 | C_16_H_12_O_5_ | 284.06848 | 284.06867 | -0.7 | [M-H] ^−^ | 283.06137 | 268.03772; 117.03318; 240.04193; 239.03467; 151.00253; 148.01540 | FON | [2] |
| 63 | Dihydroxy- octadecadien oic acid | 29.55 | C_18_H_32_O_4_ | 312.23006 | 312.23032 | -0.8 | [M-H] ^−^ | 311.22305 | 183.01128; 223.16995; 87.04373; 58.00463; 146.96013; 216.00926 | FA | [3] |
| 64 | Epiisorosmanol | 29.78 | C_20_H_26_O_5_ | 346.17803 | 346.17809 | -0.2 | [M-H] ^−^ | 345.17100 | 301.18097; 302.18439; 283.17041; 271.17010; 259.17151; 191.16194 | DT | [2] |
| 65 | Galdosol | 30.56 | C_20_H_24_O_5_ | 344.16238 | 344.1624 | -0.1 | [M-H] ^−^ | 343.15509 | 299.16528; 300.16867; 216.07855; 243.10216; 136.18471; 139.70206 | DT | [3] |
| 66 | Unknown | 30.63 | C_19_H_28_O_4_ | 320.19876 | 320.19885 | -0.3 | [M-H] ^−^ | 319.19150 | 181.08612; 138.03111; 163.07544; 153.09103; 193.08612; 125.09600 | [-] | [-] |
| 67 | Miltipolone | 30.66 | C_19_H_24_O_3_ | 300.17255 | 300.17269 | -0.5 | [M-H] ^−^ | 299.16530 | 284.14169; 243.10254; 256.11060; 230.09451; 216.07896; 150.98619 | DT | [2] |
| 68 | Ferruginol | 30.93 | C_19_H_26_O_2_ | 286.19328 | 286.19335 | -0.2 | [M-H] ^−^ | 285.18613 | 229.12297; 201.09187; 214.09935; 230.12637; 270.16174; 101.29562 | DT | [2] |
| 69 | Carnosol | 30.93 | C_20_H_26_O_4_ | 330.18311 | 330.18326 | -0.4 | [M-H] ^−^ | 329.17580 | 285.16604; 201.09169; 270.16211; 199.42516; 55.78112 | DT | [1] |
| 70 | Rosmadial | 31.22 | C_20_H_24_O_5_ | 344.16238 | 344.16245 | -0.2 | [M-H] ^−^ | 343.15524 | 315.16028; 287.16531; 316.16357; 299.16050; 288.16824; 269.15402 | DT | [2] |
| 71 | Feruginidin | 31.31 | C_22_H_30_O_5_ | 374.20933 | 374.20948 | -0.4 | [M-H] ^−^ | 373.20220 | 283.17047; 284.17377; 329.17587; 227.10751; 268.14688 | ST | [2] |
| 72 | Rosmadial isomer | 31.40 | C_20_H_24_O_5_ | 344.16238 | 344.16245 | -0.2 | [M-H] ^−^ | 343.15520 | 299.16534; 271.17044; 281.15497; 284.14145; 315.15936; 243.10190 | DT | [2] |
| 73 | 13-hydroxy-9,11-octadecadienoic acid | 31.46 | C_18_H_32_O_3_ | 296.23515 | 296.23537 | -0.8 | [M-H] ^−^ | 295.22803 | 277.21738; 195.13837; 171.10176; 183.01137; 113.09594; 96.95870 | FA | [3] |
| 74 | Rosmaridiphenol | 31.50 | C_20_H_28_O_3_ | 316.20385 | 316.20394 | -0.3 | [M-H] ^−^ | 315.19672 | 285.18610; 286.18939; 201.09186; 214.10066; 133.44701; 54.95501 | DT | [2] |
| 75 | Sugiol | 31.56 | C_20_H_28_O_2_ | 300.20893 | 300.20916 | -0.8 | [M-H] ^−^ | 299.20184 | 227.10724; 184.01442; 283.16898; 269.15442 | DT | [2] |
| 76 | Rosmanol - CO_2_ | 31.61 | C_19_H_26_O_3_ | 302.18820 | 302.18840 | -0.7 | [M-H] ^−^ | 301.18110 | 258.12607; 283.17023; 259.12952; 273.18619; 217.12308; 161.05977 | [DT] | [2] |
| 77 | Undecylbenzenesulfonic acid | 31.65 | C_17_H_28_O_3_S | 312.17255 | 312.17632 | 1.3 | [M-H] ^−^ | 311.16904 | 183.01132; 184.01801; 216.00925; 170.00372; 79.95546; 197.02739 | BSA | [3] |
| 78 | Carnosic acid | 31.82 | C_20_H_28_O_4_ | 332.19876 | 332.19889 | -0.4 | [M-H] ^−^ | 331.19153 | 287.20175; 288.20511; 244.14687; 245.15123; 111.32819 | DT | [1] |
| 79 | Taxodione | 32.04 | C_20_H_26_O_3_ | 314.18820 | 314.18859 | -1.26 | [M-H] ^−^ | 313.18127 | 298.15753; 299.16086; 65.01315; 64.00530; 183.01129; 184.01494 | DT | [2] |
| 80 | 12-Metoxy-carnosic acid | 32.23 | C_21_H_30_O_4_ | 346.21441 | 346.21450 | -0.3 | [M-H] ^−^ | 345.20721 | 286.19385; 287.19724; 301.21689; 302.22037; 215.10802; 182.11192 | DT | [2] |
| 81 | Steviol | 32.33 | C_20_H_30_O_3_ | 318.21950 | 318.21962 | -0.4 | [M-H] ^−^ | 317.21231 | 179.10690; 299.20175; 180.11034; 300.20493; 164.08336; 165.09119 | DT | [2] |
| 82 | Cryptotanshinone | 32.49 | C_19_H_20_O_3_ | 296.14125 | 296.14122 | 0.1 | [M+H]^+^ | 297.1486 | 269.15381; 171.08064; 199.07546; 227.10693; 270.15729; 69.07076 | DT | [2] |
| 83 | Nemorosin | 32.61 | C_20_H_27_O_4_ | 331.19094 | 331.19120 | -0.8 | [M-H] ^−^ | 330.18289 | 287.20175; 288.20517; 244.14674; 245.15047; 301.18100; 302.18613 | DT | [2] |
| 84 | Royleanone | 32.62 | C_20_H_28_O_3_ | 316.20385 | 316.20413 | -0.9 | [M-H] ^−^ | 315.19681 | 287.20166; 288.20502; 244.14635; 269.19070; 272.17792 | DT | [2] |
| 85 | Carnosol-CO_2_ | 32.71 | C_19_H_26_O_2_ | 286.19328 | 286.19344 | -0.6 | [M-H] ^−^ | 285.18625 | 269.15366; 270.16229; 255.13872; 257.15555; 201.09184 | DT | [3] |
| 86 | Carnosic acid derivative | 32.73 | C_30_H_40_O_6_ | 496.28228 | 496.28298 | 1.0 | [M-H] ^−^ | 495.27567 | 315.16034; 359.15012; 287.20114; 451.28397; 331.19199; 468.28522 | DT | [-] |
| 87 | 1-Oxoferruginol | 32.78 | C_20_H_28_O_2_ | 300.20893 | 300.2092 | -0.9 | [M-H] ^−^ | 299.20193 | 243.13882; 244.14226; 184.01437; 283.16946 | DT | [2] |
| 88 | Carnosic acid-CO_2_ | 32.86 | C_19_H_28_O_2_ | 288.24532 | 288.20918 | 0.9 | [M-H] ^−^ | 287.20197 | 272.17828; 194.02170; 150.03084 | DT | [3] |
| 89 | Salviol | 33.18 | C_20_H_30_O_2_ | 302.22458 | 302.22483 | -0.8 | [M-H] ^−^ | 301.21740 | 286.19324; 285.18536; 177.09138 | DT | [2] |
| 90 | Grandidone D | 33.26 | C_40_H_48_O_8_ | 656.33492 | 656.33555 | -1.0 | [M-H] ^−^ | 655.32830 | 567.34814; 611.33795; 612.34155; 298.15744; 283.17029; 112.98429 | DT | [2] |
| 91 | 24-Hydroxyglabrolide | 33.28 | C_30_H_44_O_5_ | 484.32002 | 484.31926 | 0.80 | [M-H] ^−^ | 483.3117 | 465.30099; 481.29568; 439.32187; 286.19391; 284.17853; 301.18063 | TT | [3] |
| 92 | Micromeric acid | 33.44 | C_30_H_46_O_3_ | 454.34470 | 454.34480 | -0.2 | [M+H]^+^ | 455.35190 | 189.16415; 203.18002; 119.08588; 95.08625; 409.34769; 391.33643 | TT | [2] |
| 93 | Salvadione C | 33.46 | C_30_H_40_O_5_ | 480.28758 | 480.28789 | -0.7 | [M-H] ^−^ | 479.28070 | 435.29050; 283.17035; 299.16550; 343.15524; 329.17575; 141.05458 | TT | [2] |
| 94 | Horminone | 33.77 | C_20_H_28_O_4_ | 332.19876 | 332.19908 | -1.0 | [M-H] ^−^ | 331.19183 | 287.20175; 288.20514; 244.14668; 112.98432; 71.92699; 207.02979 | DT | [2] |
| 95 | Salvadione A | 33.78 | C_30_H_42_O_4_ | 466.30831 | 466.30845 | -0.3 | [M-H] ^−^ | 465.30099 | 351.23257; 378.25641; 337.21735; 323.20178; 309.18564; 209.21739 | TT | [2] |
| 96 | Betulinic acid | 34.05 | C_30_H_48_O_3_ | 456.36035 | 456.36072 | -0.8 | [M-H] ^−^ | 455.35340 | 287.20251; 301.18082; 312.42496; 113.96554; 61.98687 | TT | [2] |
| 97 | Ursolic acid | 34.18 | C_30_H_48_O_3_ | 456.36035 | 456.36069 | -0.8 | [M-H] ^−^ | 455.3534 | 229.23468; 323.25034; 210.69127; 155.42545; 136.85194 | TT | [2] |
| 98 | Methyl carnosate | 34.71 | C_21_H_30_O_4_ | 346.21441 | 346.21446 | -0.1 | [M-H] ^−^ | 345.20721 | 286.19394; 287.19730; 301.21713; 302.22064; 271.17081; 215.10826 | DT | [2] |

Classes: BSA, benzenesulfonic acids; CA, carboxylic acids; DT, diterpenoids; FA, fatty acids; FVA, flavanones; FON, flavones; FOL, flavonols; Gly, glycosides; HCU, hydroxycoumarins; JAS, jasmonates; OS, oligosaccharides; PA, phenolic acids; SI, secoiridoids; ST, sesquiterpenoids; SA, sugar acids; TT, triterpenoids. IDL – Identification confidence level: [1], identification confirmed via reference standard by MS, MS^2^ and RT data; [2], identification based on MS, MS^2^ and literature data for salvia species; [3], tentative identification based on MS and MS^2^ data.
